# 

**Published:** 1893-03

**Authors:** 


### BOOKS RECEIVED.

Syphilis and the Nervous System, being a revised reprint of the Lettsomian Lectures for 1890, delivered before the Medical Society of London. By W. R. Gowers, M. D., F. R. C. P., F. R. S., Consulting Physician to University College Hospital, Physician to the National Hospital for the paralyzed, and epileptic, etc. Price, $1.00. Philadelphia: P. Blakiston, Son & Co., 1012 Walnut street. 1892.

Fermentation, Infection, and Immunity. A new theory of these processes, which unifies their primary causation, and places the explanation of their phenomena in chemistry, biology, and the dynemics of molecular physics. By J. W. McLaughlin, M. D., Austin, Texas. Copyright, 1892. Austin, Texas: Eugene Von Boeckmann, printer and bookbinder. 1892.

Hand-book of Insanity for Practitioners and Students. By Dr. Theodore Kirchhoff, Physician to the Schleswig Insane Asylum, and Privatdocent at the University of Kiel. Illustrated with eleven plates. Octavo volume, pp. 362. New York: William Wood & Co. 1893.

Hygiene and Public Health. By Louis C. Parkes, M. D., D. P. H., London University. Fellow of the Sanitary Institute, and Member of the Board of Examiners; Lecturer on Public Health at St. George's Hospital Medical School; Medical Officer of Health and Public Analysis for the parish of Chelsea. Third edition, with illustrations, Price, $2.75. Philadelphia: P. Blakiston, Son & Co., 1012 Walnut street. 1892.

The Chronic Disorders of the Digestive Tube. By W. W. Van Valzah, A. M., M. D., formerly Demonstrator of Clinical Medicine, Jefferson Medical College. Octavo volume, pp. iv.—151. New York: J. H. Vail & Co. 1893.

Lectures on Mental Diseases, designed especially for Medical Students and General Practitioners. By Henry Putnam Stearns, A. M., M. D., Physician Superintendent of the Hartford Retreat, Lecturer on Mental Diseases in Yale University; Member of the American Medico-Psychological Association, Member of the New England Psychological Society, Honorary Member of the British Psychological Association, Honorary Member of the Boston Medico-Psychological Society, Member of the American Medical Association, etc., etc. With illustrations. Small octavo; pp. xviii.—636. Price, $3.00. Philadelphia: P. Blakiston, Son & Co., 1012 Walnut street. 1893.

Hand-book of Materia Medica, Pharmacy, and Therapeutics, including the Physiological Action of Drugs, the special Therapeutics of Disease, Official and Practical Pharmacy, and Minute Direction for Prescription Writing. By Samuel O. L. Potter, A. M., M. D., M. R. C. P., Lond., Professor of the Theory and Practice of Medicine in the Cooper Medical College, of San Francisco; Visiting Physician to St. Luke's Hospital; Author of Quiz Compends of Anatomy and Materia Medica, An Index of Comparative Therapeutics, and A Study of Speech and Its Defects. Formerly A. A. Surgeon, U. S. Army, and Brigade-Surgeon, N. G. of California. Fourth edition, revised. Octavo volume, pp. xii. —781. Price, $4.00. Philadelphia: P. Blakiston, Son & Co., 1012 Walnut street. 1893.

A System of Genito-Urinary Diseases, Syphilology and Dermatology, by various authors. Edited by Prince A. Morrow, A. M., M. D., Clinical Professor of Genito-Urinary Diseases; formerly Lecturer on Dermatology in the University of the City of New York; Surgeon to Charity Hospital, etc. With illustrations. In three large 12mo volumes. Volume I., Genito-Urinary Diseases. Sold only by subscription.

Drifting. By Vigilans. Denver: Printed and for sale by the Chain & Hardy Co. 1892. Price, 50c. Octavo, pp. 313.

NOTICE TO CONTRIBUTORS.—We are glad to receive contributions from every one who knows anything of interest to the profession. Articles designed for publication in the JOURNAL should be handed in before the first day of the month. The Editors are not responsible for the views or opinions of contributors. All communications should be addressed to the Managing Editor, 284 FRANKLIN ST., BUFFALO, N. Y.

